# Rapid and Unpredictable Shifts in Perceived Pleasantness of Continuous Affective Touch

**DOI:** 10.3390/bs15060712

**Published:** 2025-05-22

**Authors:** Anne Schienle, Carina Schlintl, Arved Seibel

**Affiliations:** Institute of Psychology, University of Graz, Universitätsplatz 2, 8010 Graz, Austria

**Keywords:** affective touch, pleasantness, habituation, childhood touch, sympathy, depression, somatization

## Abstract

Affective touch (stroking the skin at velocities between 1 and 10 cm/s) is generally perceived as pleasant. However, this pleasant sensation diminishes with continuous stimulation over several minutes, with substantial individual variability in the habituation process. This study aimed to identify individual characteristics associated with the decline in the hedonic value of prolonged affective touch. Eighty-one female participants (mean age = 26 years) received continuous stroking on their forearms for 10 min at two distinct velocities: 3 cm/s (affective touch) and 30 cm/s (nonaffective touch). Every 100 s, participants rated the perceived pleasantness of the stimulation. Regression analyses were conducted to examine whether participants’ age, attitude toward touch by an unfamiliar person, recalled positive touch experiences during childhood, sympathy toward the toucher, reported symptoms of anxiety, depression, or somatization, and order of touch conditions predicted changes in their responses. On average, the perceived pleasantness of touch declined over time. The extent of the decline and individual variability in pleasantness ratings were not significantly associated with the selected predictors. However, higher overall ratings of affective touch pleasantness were linked to greater sympathy toward the toucher, lower levels of depression and somatization, and a lower frequency of recalled positive touch experiences during childhood. Affective touch was perceived as more pleasant when it was preceded by the nonaffective touch condition, compared to when the order was reversed. Order effects, the rapid decline, and substantial individual variability in the perceived pleasantness of prolonged affective touch should be considered in both research and therapeutic applications.

## 1. Introduction

Soft stroking of the skin at velocities ranging between 1 and 10 cm/s is generally perceived as pleasant (Löken et al., 2009). This form of tactile stimulation referred to as ‘affective touch’ activates C-tactile (CT) afferents, which transmit their input to specific areas of the brain, such as the insula and the orbitofrontal cortex. These regions play a crucial role in decoding the emotional value of a stimulus (McGlone et al., 2014). Affective touch serves as a vital channel for social communication, fostering attachment, and bonding in relationships such as those between parents and children, friends, or romantic partners. It also plays a key role in regulating emotions and pain, making it a crucial factor in promoting overall health and well-being.

When CT-optimal stroking is repeated over several minutes, the subjective experience of this type of tactile stimulation undergoes temporal changes. On average, the positive valence of affective touch diminishes. This habituation process has been systematically investigated for the first time by Triscoli et al. (2014). In two experiments, the authors delivered brush stroking to the forearms of the participants for 50 min at three different velocities (affective touch: 3 cm/s; nonaffective touch: 0.3 cm/s, 30 cm/s). Participants rated the pleasantness (liking) and wanting (the wish to be further exposed to the same stimulus) of the tactile stimulation after each stroke. In experiment 1, pleasantness and wanting for affective touch decreased over repetitions. In contrast, nonaffective touch showed no decrease in ratings over time. In experiment 2, reductions in pleasantness and wanting were observed over repetitions for both affective and nonaffective touch. The authors referred to the observed phenomenon as ‘touch satiety’.

In a subsequent study by the same authors (Triscoli et al., 2017), participants received either 35 min of brush stroking (affective touch: 3 cm/s) or vibrational stimulation on their forearms. It was found that the pleasantness of being touched continuously decreased over time for both conditions. At the end of the experiment, affective touch was even perceived as negative on average. In contrast, touch intensity was rated as being temporally stable by both groups. Very similarly, Sailer et al. (2016) administered affective touch (3 cm/s) for 40 min. Pleasantness ratings for touch (assessed every two minutes) also continuously decreased over time.

In another investigation, Bendas et al. (2021) applied continuous affective touch at a velocity of 3 cm/s to participants’ forearms across two successive experimental sessions, each lasting 30 min. On average, the hedonic evaluation of affective touch exhibited a decline over time. Nevertheless, substantial interindividual variability was observed, with some participants demonstrating decreases and others increases in perceived pleasantness throughout the experimental period. A key methodological limitation identified by the authors was the absence of a tactile control condition, which constrained the ability to ascribe observed effects specifically to affective touch. This limitation was addressed in the present study through the inclusion of a control condition with nonaffective touch.

Furthermore, Bendas et al. (2021) reported that patterns of habituation to affective touch were neither significantly associated with individual differences in personality traits (reward sensitivity, openness to experience, conscientiousness, extraversion, agreeableness, and neuroticism) nor with physiological indicators (electrocardiographic or facial electromyographic responses). Consequently, factors associated with affective touch habituation remain to be elucidated. Identifying such factors constituted a central objective of the current investigation.

In contrast, several variables that are correlated with the perceived overall pleasantness of affective touch (averaged pleasantness across a stimulation interval) have already been identified. It is known that context factors shape the hedonic tone of this type of skin stimulation (for an overview see Ellingsen et al., 2016). For example, characteristics of the toucher and toucher-related information (e.g., concerning sympathy/strength of the emotional bond, physical characteristics, and intentions) are associated with the evaluation of affective touch (Suvilehto et al., 2015; Ellingsen et al., 2016; Novembre et al., 2021). Moreover, age and experience with affective touch are connected with the reported valence for affective touch. Across the lifespan, affective touch becomes more positive, on average (McIntyre et al., 2021). This effect is particularly prominent in older individuals, who are touched less frequently in their daily lives than younger individuals (e.g., Schlintl & Schienle, 2024). Moreover, adverse childhood experiences, such as neglect or abuse, are associated with reduced pleasure or even discomfort from affective touch (Trotter et al., 2018).

The sex of the toucher as well as the sex of the person being touched also plays a role in affective touch evaluation. In an online survey (Schirmer et al., 2022), female participants reported preferring touch with other females, expressed more touch comfort with less familiar or unknown individuals, and felt more comfortable receiving touch to the forearm than males. The meta-analysis by Russo et al. (2020) indicated that females gave higher pleasantness ratings than males for affective touch.

Finally, previous research has indicated that emotional reactions to tactile stimuli are associated with diagnoses of specific mental health conditions (e.g., Croy et al., 2016; He et al., 2021; Perini et al., 2021). For example, Croy et al. (2016) compared a group of patients from an outpatient clinic (main diagnoses: affective/somatoform/personality disorders) with healthy controls. The participants received tactile stimulation at their forearms with different velocities (0.3/1/3/10/30 cm/s). The patients experienced touch as generally less pleasant than the healthy controls. This effect was especially pronounced in the patients with personality disorders.

The mentioned studies point to alterations in affective touch processing in specific mental health conditions. Whether specific symptom dimensions are linked with altered affective touch habituation has not been investigated thus far. Therefore, the present study analyzed associations between reported symptoms of anxiety, somatization, and depression and reductions in the perceived pleasantness of affective touch over time. Participants received both affective and nonaffective touch continuously administered via a soft brush for over 10 min. They rated the pleasantness of the tactile stimulation to their forearms every 100 s. Regression analyses were computed to identify possible associations between the average pleasantness ratings for affective touch (criterion 1) and the change in pleasantness ratings from the beginning to the end of the experiment (criterion 2) with the selected predictors (anxiety, depression, somatization, age, sympathy with the toucher, attitude towards being touched by a stranger, order of touch conditions, recalled positive touch experiences in childhood).

## 2. Materials and Methods

### 2.1. Participants

Participants were recruited via mass mailings to students at the university, social media, and flyers placed at counseling centers. Informed consent was obtained from all participants. The inclusion criteria were a minimum age of 18 years and female sex. All participants were right-handed. The sample was restricted to females due to sex-related variance in affective touch evaluation (Russo et al., 2020). Exclusion criteria were skin changes at the forearm and diagnoses of neurological diseases that impact touch processing.

The final sample consisted of 81 females (average age: M = 26.11 years, SD = 7.83, range: 18–54 years). A power analysis using G*Power version 3.1.9.7 indicated that a sample size of 80 was required for a multiple regression with seven^1^ predictors, assuming a medium effect size (f^2^ = 0.20), power of 0.80, and an alpha level of 0.05.

### 2.2. Procedure

Participants placed their left forearm on a table and were instructed to close their eyes. A trained female experimenter, seated at a 90-degree angle, applied skin stroking using a soft bristle brush (Bipa). Two conditions were tested—Condition A (3 cm/s) and Condition B (30 cm/s)—with the order counterbalanced across participants and a 20 min break between conditions. Stroking speed was controlled using a metronome app played through headphones. Each condition lasted 10 min, with continuous stroking throughout. During each condition, participants received an acoustic signal every 100 s (six times in total), prompting them to open their eyes and rate the stimulation in terms of pleasantness and intensity (scale: 0 = low, 100 = high). Ratings were given by moving a marker along a horizontal bar on a computer screen using the right hand and a computer mouse. After each condition, participants also rated their level of sympathy toward the experimenter on a visual analogue scale from 0 to 100 (100 = very sympathetic).

### 2.3. Questionnaires

(a) The Brief Symptom Inventory (Spitzer et al., 2011) consists of 18 items covering three symptom dimensions: Depression (e.g., feeling lonely/blue), Anxiety (e.g., feeling afraid), and Somatization (e.g., pain/dizziness). The presence of symptoms is rated on a 5-point scale (0  =  not at all; 4  =  very intense). For each of the scales, a sum score is calculated. Cronbach’s alpha for the subscales ranged from 0.79 to 0.85.
(b) The subscales Attitude to Unfamiliar Touch (AUT; 5 items) and Childhood Touch (ChT; 9 items) are part of the Touch Experiences and Attitudes Questionnaire (TEAQ, Trotter et al., 2018). The two subscales measure an individual’s tendency to feel comfortable with physical touch from unfamiliar people (e.g., “I have to know someone quite well to enjoy a hug from them.”) and experiences with affective touch during childhood (e.g., “My parents were not very physically affectionate towards me during my childhood”). Participants rate each statement on a 5-point Likert scale ranging from 1 “Disagree strongly” to 5 “Agree strongly”. For the AUT subscale, the Cronbach’s alpha in the present sample was 0.77, and for the ChT subscale it was 0.90. For both subscales, a mean score was calculated with higher values indicating greater comfort with unfamiliar touch (AUT) and more positive childhood touch experiences (ChT).

### 2.4. Statistical Analyses

Two analyses of variance (ANOVAs) were computed to examine the effects of touch condition (affective vs. nonaffective) and time (six rating points) on perceived touch pleasantness and intensity (SPSS, version 29). Post hoc pairwise comparisons were performed to assess changes in ratings between consecutive time points (as indicators of continuous habituation). We report Greenhouse–Geisser corrected results when assumptions of sphericity were violated.

In addition, a multiple regression analysis was conducted to investigate the association between overall pleasantness of affective touch (criterion 1) and the assessed predictors: participants’ age, sympathy towards the toucher, order of touch conditions, participants’ attitude to unfamiliar touch, childhood touch, and the three BSI scales. Criterion 1 was calculated as the average of the six pleasantness ratings for stroking at a velocity of 3 cm/s. A second multiple regression analysis was conducted to investigate the association between changes in pleasantness of affective touch (criterion 2) and the assessed predictors. Criterion 2 was calculated by subtracting the first pleasantness rating in the affective touch condition (stroking velocity: 3 cm/s) from the last rating. Negative values indicate habituation.

Finally, exploratory regression analyses were carried out for the criterion pleasantness of touch in the nonaffective touch condition and the criterion intensity of perceived touch (for both touch conditions).

## 3. Results

### 3.1. Analysis of Variance—Pleasantness

**Figure 1:**
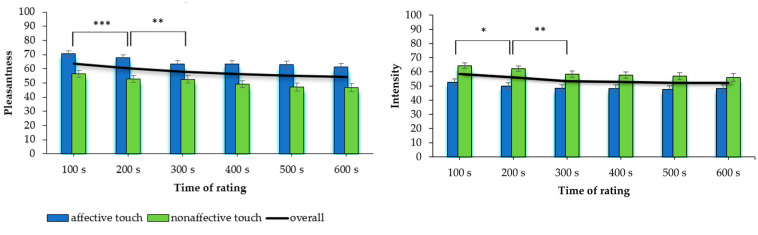
Mean ratings (standard errors) for perceived pleasantness and intensity of touch in (non)affective touch conditions and across conditions (overall). Note: Pairwise comparisons for consecutive time points; * *p* < 0.05, ** *p* < 0.01, *** *p* < 0.001.

The ANOVA identified a significant main effect of touch condition (F(1, 80) = 46.11, *p* < 0.001, pη^2^ = 0.37). Perceived pleasantness was higher in the affective touch condition (M = 64.91, SD = 18.87) compared to the nonaffective touch condition (M = 50.81, SD = 20.96; Figure 1). The effect of time was statistically significant as well (F(2.19, 175.49) = 14.56, *p* < 0.001, pη^2^ = 0.15; Figure 1). The interaction effect was not significant (F(3.21, 257.15) = 1.14, *p* = 0.333, pη^2^ = 0.01).

Paired *t*-tests showed a significant decrease in pleasantness from time point 1 (M = 63.62, SD = 16.68) to time point 2 (M = 60.30, SD = 18.29, t(80) = 3.43, *p* < 0.001, d = 0.38), and from time point 2 to time point 3 (M = 57.94, SD = 19.06, t(80) = 2.70, *p* = 0.008, d = 0.30). All other comparisons of consecutive time points were not significant (*p* > 0.050).

### 3.2. Analysis of Variance—Intensity

The main effect of touch condition was significant (F(1, 80) = 20.93, *p* < 0.001, pη^2^ = 0.21). Perceived intensity was higher in the nonaffective touch condition (M = 59.29, SD = 16.86) compared to the affective touch condition (M = 49.15, SD = 20.17). The main effect of time was significant (F(2.25, 180.12) = 6.96, *p* < 0.001, pη^2^ = 0.08). The interaction effect was not significant (F(2.51, 200.64) = 0.92, *p* = 0.419, pη^2^ = 0.01).

Paired *t*-tests showed a significant difference in perceived intensity between time point 1 (M = 58.48, SD = 16.04) and time point 2 (M = 56.08, SD = 15.32, t(80) = 2.41, *p* = 0.018, d = 0.27; Figure 1), as well as time point 2 and time point 3 (M = 53.40, SD = 16.96, t(80) = 2.70, *p* = 0.008, d = 0.30). All other comparisons of consecutive time points were not significant (*p* ≥ 0.050).

### 3.3. Correlation Analyses

Somatization scores were negatively associated with the average pleasantness ratings during the affective touch condition, while sympathy with the toucher was positively correlated (Table 1). None of the selected variables was significantly correlated with the average intensity of affective touch. Pleasantness and intensity ratings were independent of each other. A table with correlation coefficients for the nonaffective touch condition is provided in the Supplementary Material (Table S1).

### 3.4. Regression Analysis 1: Overall Pleasantness of Affective Touch

The multiple regression model was statistically significant (F(8, 66) = 3.28, *p* = 0.003), explaining 28% of the variance in the criterion variable (Table 2). Among the predictors, sympathy with the toucher, order of touch conditions, somatization, depression, and childhood touch were statistically significant. Sympathy showed a positive relation with perceived pleasantness (B = 0.53, SE = 0.15, t = 3.44, *p* = 0.001). Furthermore, experiencing affective touch as the first condition was associated with lower pleasantness ratings compared to the reversed order (B = −8.24, SE = 3.94, t = −2.09, *p* = 0.040). Somatization (B = −1.24, SE = 0.54, t = −2.30, *p* = 0.025), depression (B = −0.97, SE = 0.47, t = −2.07, *p* = 0.043), and frequency of recalled positive childhood touch had a negative association with the criterion (B = −5.38, SE = 2.29, t = −2.35, *p* = 0.022).

The model for nonaffective touch was not statistically significant (F(8, 66) = 1.65, *p* = 0.127; Table S2).

### 3.5. Regression Analysis 2: Changes in Pleasantness Ratings

Perceived pleasantness during the affective touch condition decreased from the first rating (M = 70.81, SD = 18.51) to the last rating (M = 61.23, SD = 23.39, t(80) = 4.12, *p* < 0.001, d = 0.46). The multiple regression analysis was not statistically significant (F(8, 66) = 1.11, *p* = 0.370; Table S3).

In the nonaffective touch condition, perceived pleasantness decreased from the first (M = 56.43, SD = 20.53) to the last rating (M = 46.88, SD = 26.00, t(80) = 3.63, *p* < 0.001, d = 0.40). The corresponding multiple regression analysis was not significant (F(8, 66) = 0.77, *p* = 0.634; Table S4).

### 3.6. Exploratory Regression Analyses for Perceived Intensity of Touch

The analyses did not yield statistically significant findings, neither for the criterion overall intensity of perceived affective touch (F(8, 66) = 0.80, *p* = 0.604) and nonaffective touch (F(8, 66) = 0.54, *p* = 0.826) nor changes in perceived intensity in the affective (F(8, 66) = 0.24, *p* = 0.982) and nonaffective touch conditions (F(8, 66) = 0.81, *p* = 0.599) (Tables S5–S8).

### 3.7. Individual Response Patterns

**Figure 2:**
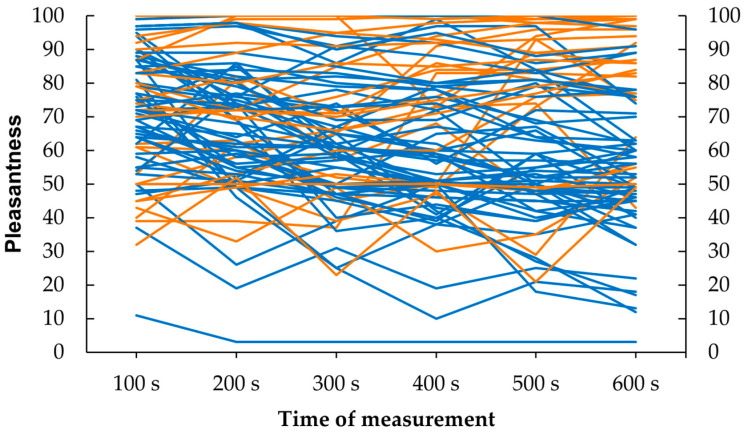
Individual temporal changes in pleasantness ratings for affective touch. Note: Habituation responses (where rating at 600 s is lower than at 100 s) are shown in blue; increases/no changes in pleasantness ratings (600 s vs. 100 s) are shown in orange.

Individual changes in pleasantness ratings over time during the affective touch condition revealed a complex pattern as illustrated in Figure 2. While approximately two-thirds of participants reported a decrease in perceived pleasantness from the first to the last rating, one third showed no decrease or even an increase in pleasantness. Additionally, many participants exhibited fluctuations in their ratings, with both increases and decreases occurring over time. To account for this variability, the variance in ratings (calculated as the sum of the squared changes between consecutive time points, divided by the number of changes) was used as an additional criterion in an exploratory regression analysis. However, none of the selected predictors (age, attitude to unfamiliar touch, childhood touch, sympathy toward the toucher, order of touch condition, anxiety, somatization, or depression) was found to predict individual differences in rating variance (Table S9).

## 4. Discussion

This study focused on the evaluation of continuous affective and nonaffective touch administered to the forearm of the participants for 10 min. Ratings for perceived pleasantness and intensity of the tactile stimulation were assessed every 100 s.

We identified findings concerning perceived overall pleasantness of affective touch that are consistent with previous research: affective touch was rated as more pleasant than nonaffective touch (for a review, see Cruciani et al., 2021), and greater sympathy toward the toucher was associated with greater perceived pleasantness of affective touch (Ellingsen et al., 2016).

Additionally, fewer recalled instances of positive touch during childhood (e.g., hugging or holding hands with family members) were negatively correlated with the perceived valence of affective touch. This may suggest that individuals who experienced less affective touch within their families may develop a stronger desire for this type of tactile stimulation. A similar phenomenon was observed during the COVID-19 outbreak, where reduced touch experiences were linked to an increased desire for and heightened pleasantness of being touched (e.g., Ujitoko et al., 2022).

In the present study, higher scores on the BSI depression scale were associated with lower ratings of affective touch pleasantness. This finding partially aligns with previous research showing that individuals with elevated depression scores exhibited a less positive attitude toward social touch (Triscoli et al., 2019) or reported reduced pleasantness for both affective and nonaffective touch (Tinker et al., 2023). In the latter study, participants watched videos of social touch and rated the statement, “How pleasant would it be to be touched like this?” Thus, their ratings reflected imagined rather than actual touch experiences, in contrast to the present experiment, where participants received real touch stimuli. Additionally, we observed an association between somatization and affective touch pleasantness. The BSI somatization scale measures how often someone is troubled by general bodily discomfort without a clear physical cause. Physical discomfort might be linked with heightened touch sensitivity, and if tied to emotional stress, it could manifest as tactile overstimulation.

Concerning habituation, we found that the perceived pleasantness and intensity of strokes to the forearm decreased over time when applied at a slow as well as high speed, partially replicating the research by Triscoli et al. (2014). On average, habituation occurred quickly with significant reductions in perceived pleasantness/intensity within the first two minutes of tactile stimulation.

Further, participants varied widely in how pleasant the touch was perceived, both initially and over the course of the study. While we found habituation effects on the group level, individual rating patterns diverged markedly in both conditions. This is in line with the research by Bendas et al. (2021), who also observed a high degree of interpersonal variation during the course of a brush-stroking paradigm. In their study, only 20 out of 47 participants exhibited a linear decrease in pleasantness ratings for continuous affective touch. Taken together, affective touch habituation seems to be a robust phenomenon on the group level, but not on the individual level.

For touch habituation (total magnitude and variation in pleasantness ratings), we were not able to identify any significant associations with the selected variables, including age of the participants, their sympathy with the toucher, and their touch attitude and experience. Notably, none of the dimensions of the brief symptom inventory (anxiety, depression, somatization) was able to predict temporal changes in the perceived pleasantness of affective touch. Other studies also failed at establishing a connection of trait variables and touch habituation (Bendas et al., 2021). Therefore, it remains an open question as to what extent trait (i.e., temporally stable) variables play a role in affective touch habituation. Yet, potentially interesting variables (e.g., attachment style) and disorders (e.g., borderline personality disorder, autism-spectrum disorders) remain to be explored within the context of touch habituation.

Interestingly, the order of touch conditions emerged as a significant predictor of the overall pleasantness of affective touch. This finding highlights the influence of contextual factors on touch processing (Sailer & Leknes, 2022). Touch experiences are embedded within a broader contextual framework, which may be processed either implicitly or explicitly. Experiencing fast nonaffective touch followed by slow touch at a CT-optimal velocity may have created a positive contrast effect, resulting in higher pleasantness ratings.

Our findings should be considered in light of several limitations. Due to the experimental setup, ecological validity is limited. Stimulation was applied monotonously using a brush to the participants’ forearms (Kress et al., 2011). In other studies, stroking was performed by hand (Bendas et al., 2021), which is more natural but may have elicited apprehensions among participants, such as concerns about hygiene. (The current study was conducted shortly after the COVID-19 pandemic.)

Ratings were only collected every 100 s. In future studies, assessments could be conducted earlier and more frequently to capture more nuanced responses over time (Triscoli et al., 2014).

Participant–researcher dynamics were only covered incompletely. For example, experimenter-related evaluations were restricted to sympathy. However, several other factors, such as perceived friendliness or sexual orientation/preference could also influence responses (Ellingsen et al., 2016). Lastly, our results cannot be generalized to males.

We would also like to point out an asset of the present study. Due to the inclusion of a tactile control condition in combination with both pleasantness and intensity ratings, we were able to detect findings that are specific for affective touch pleasantness.

## 5. Conclusions

This study enhances our understanding of how affective touch perception changes over time. The rapid alterations and pronounced individual variability in the perceived pleasantness of prolonged touch must be considered in both research and therapeutic contexts. It cannot be assumed that affective touch will remain consistently and sufficiently pleasant for an individual over several minutes.

## Figures and Tables

**Table 1:** Descriptive statistics and correlations for affective touch.

	M (SD)	1	2	3	4	5	6	7
Affective touch								
Pleasantness (1)	64.91 (18.87)							
Intensity (2)	49.15 (20.17)	0.11						
Sympathy (3)	86.57 (13.25)	0.33 *	0.10					
BSI Somatization (4)	5.05 (4.90)	−0.25 *	0.03	−0.01				
BSI Depression (5)	7.96 (5.59)	−0.20	0.05	0.08	0.49 **			
BSI Anxiety (6)	7.72 (5.04)	−0.17	0.09	−0.08	0.63 **	0.63 **		
TEAQ AUT (7)	3.00 (0.88)	0.12	0.09	0.15	−0.32 **	−0.23 *	−0.24 *	
TEAQ ChT (8)	3.54 (0.99)	−0.14	0.07	−0.02	−0.13	−0.21	0.03	0.20

Note. pleasantness/intensity ratings averaged across six ratings; sympathy with toucher measured during the specific touch condition; BSI (Brief Symptom Inventory); TEAQ (Touch Experiences and Attitudes Questionnaire): AUT (Attitudes to Unfamiliar Touch), ChT (Childhood Touch); * *p* < 0.05; ** *p* < 0.001.

**Table 2:** The multiple regression analysis for overall pleasantness in the affective touch condition as the criterion.

						95% Confidence Interval for B	
	R^2^	B	SE	T	*p*	LB	UB
	0.28						
Intercept		69.87	2.79	25.06	<0.001	64.30	75.44
Age		−0.26	0.28	−0.94	0.353	−0.83	0.30
**Sympathy**		0.53	0.15	3.44	0.001	0.22	0.83
**Condition order**		−8.24	3.94	−2.09	0.040	−16.09	−0.38
TEAQ_AUT		−0.45	2.40	−0.19	0.850	−5.25	4.34
**TEAQ_ChT**		−5.38	2.29	−2.35	0.022	−9.95	−0.81
BSI-18 anxiety		1.03	0.60	1.70	0.094	−0.18	2.23
**BSI-18 somatization**		−1.24	0.54	−2.30	0.025	−2.32	−0.16
**BSI-18 depression**		−0.97	0.47	−2.07	0.043	−1.91	−0.03

Note. Pleasantness ratings averaged across six ratings; order: if nonaffective condition was completed first, this was coded with 0/reversed order: 1; sympathy with toucher was measured during affective touch condition; BSI (Brief Symptom Inventory); TEAQ (Touch Experiences and Attitudes Questionnaire): AUT (Attitudes to Unfamiliar Touch), ChT (Childhood Touch); variables are mean-centered; predictor names in bold indicate significance at *p* < 0.05.

## Data Availability

The raw data supporting the conclusions of this article will be made available by the authors on request.

## Note

1 An eighth predictor was suggested by a reviewer.

## Supplementary Materials

The following supporting information can be downloaded at https://www.mdpi.com/article/10.3390/bs15060712/s1, Table S1: Descriptive statistics and correlations for nonaffective touch; Table S2: Multiple regression analysis for overall pleasantness in the nonaffective touch condition as the criterion; Table S3: Multiple regression analysis for the pleasantness change in the affective touch condition as the criterion; Table S4: Multiple regression analysis for the pleasantness change in the nonaffective touch condition as the criterion; Table S5: Multiple regression analysis for overall intensity in the affective touch condition as the criterion; Table S6: Multiple regression analysis for overall intensity in the nonaffective touch condition as the criterion; Table S7: Multiple regression analysis for the intensity change in the affective touch condition as the criterion; Table S8: Multiple regression analysis for the intensity change in the nonaffective touch condition as the criterion; Table S9: Multiple regression analysis for the variance of pleasantness in the affective touch condition as the criterion.
