# Supplementary material for: Rapid and Unpredictable Shifts in Perceived Pleasantness of Continuous Affective Touch
Source: Behav Sci (Basel). 2025 May 22;15(6):712. doi: 10.3390/bs15060712 (PMC12189115; doi:10.3390/bs15060712)
Supplement: Supplementary file 1 [file behavsci-15-00712-s001.zip › behavsci-3584834-supplementary.pdf]

Supplementary Material

Table S1. Descriptive statistics and correlations for nonaffective touch

Descriptive Statistics and Correlations

	M (SD)	1	2	3	4	5	6	7
Pleasantness. NAT (1)	50.81(20.96)							
Intensity. NAT (2)	59.29 (16.86)	-.07						
Sympathy (3)	85.95 (13.09)	.19	.04					
BSI Somatization (4)	5.05 (4.90)	-.12	-.01	.05				
BSI Depression (5)	7.96 (5.59)	-.17	.01	.20	.49**			
BSI Anxiety (6)	7.72 (5.04)	-.13	-.01	.04	.63**	.63**		
TEAQ AUT (7)	3.00 (0.88)	.20	.01	.10	-.32**	-.23*	-.24*	
TEAQ ChT (8)	3.54 (0.99)	-.13	-.06	-.03	-.13	-.21	.03	.20

Note. NAT (nonaffective touch); pleasantness/intensity ratings averaged across six ratings; sympathy with toucher was measured during the nonaffective touch condition; BSI (Brief Symptom Inventory); TEAQ (Touch Experiences and Attitudes Questionnaire): AUT (Attitudes to Unfamiliar Touch). ChT (Childhood Touch).; *p < .05; **p < .001.

Table S2. Multiple regression analysis for overall pleasantness in the nonaffective touch condition as the criterion.

						95% Confidence Interval for B	
	R^2	B	SE	t	P	LB	UB
	.17						
Intercept		53.23	3.38	15.76	<.001	46.48	59.97
Age		0.45	0.34	1.34	.186	-0.22	1.12
**Sympathy**		0.24	0.20	1.24	.218	-0.15	0.63
**Condition order**		-4.89	4.82	-1.02	.314	-14.51	4.73
TEAQ_AUT		3.00	2.88	1.04	.301	-2.75	8.74
**TEAQ_ChT**		-3.24	2.75	-1.18	.242	-8.72	2.24
BSI-18 anxiety		0.28	0.72	0.39	.695	-1.15	1.72
**BSI-18 somatization**		-0.10	0.65	-0.16	.875	-1.39	1.19
**BSI-18 depression**		-0.91	0.57	-1.59	.116	-2.06	0.23

Note. Pleasantness ratings averaged across six ratings; order: if the nonaffective condition was completed first, this was coded with 0/ reversed order: 1; sympathy with toucher was measured during the nonaffective touch condition; BSI (Brief Symptom Inventory); TEAQ (Touch Experiences and Attitudes Questionnaire): AUT (Attitudes to Unfamiliar Touch), ChT (Childhood Touch); variables are mean-centered.

$F(8,66)=1.65$, $p=.127$

Table S3. Multiple regression analysis for the pleasantness change in the affective touch condition as the criterion

	R^2	B	SE	t	P	95% Confidence Interval for B LB	UB
	.12						
Intercept		-4.95	3.61	-1.37	.176	-12.16	2.27
Age		-0.39	0.37	-1.07	.290	-1.12	0.34
Sympathy		0.02	0.20	0.09	.929	-0.38	0.42
Condition order		-8.28	5.10	-1.62	.109	-18.47	1.90
TEAQ_AUT		-4.34	3.11	-1.40	.167	-10.56	1.87
TEAQ_ChT		-5.41	2.97	-1.82	.073	-11.33	0.52
BSI-18 anxiety		0.04	0.78	0.05	.962	-1.53	1.60
BSI-18 somatization		-0.55	0.70	-0.78	.437	-1.95	0.85
BSI-18 depression		0.18	0.61	0.29	.773	-1.04	1.39

Note. the criterion is calculated by subtracting the reported pleasantness after 100 seconds from the reported pleasantness after 600 seconds; sympathy with toucher was measured during the affective touch condition; Condition order: if the nonaffective condition was completed first. this was coded with 0/ reversed order: 1; BSI (Brief Symptom Inventory); TEAQ (Touch Experiences and Attitudes Questionnaire): AUT (Attitudes to Unfamiliar Touch). ChT (Childhood Touch); variables are mean-centered.

F(8.66)=1.11. p=.370

Table S4. Multiple regression analysis for the pleasantness change in the nonaffective touch condition as the criterion

	R^2	B	SE	t	P	95% Confidence Interval for B LB	UB
	.08						
Intercept		-7.01	4.22	-1.66	.101	-15.44	1.41
Age		0.05	0.42	0.13	.899	-0.79	0.89
Sympathy		-0.10	.24	-0.40	.689	-0.59	0.39
Condition order		-5.13	6.02	-0.85	.397	-17.15	6.89
TEAQ_AUT		-1.98	3.59	-0.55	.583	-9.16	5.19
TEAQ_ChT		-3.25	3.43	-0.95	.348	-10.09	3.60
BSI-18 anxiety		0.37	0.90	0.41	.685	-1.43	2.16
BSI-18 somatization		-1.40	0.81	-1.73	.088	-3.01	0.22
BSI-18 depression		0.70	0.72	0.97	.334	-0.73	2.13

Note. the criterion is calculated by subtracting the reported pleasantness after 100 seconds from the reported;pleasantness after 600 seconds; sympathy with toucher was measured during the nonaffective touch condition; Condition order: if the nonaffective condition was completed first. this was coded with 0/ reversed order: 1; BSI (Brief Symptom Inventory); TEAQ (Touch Experiences and Attitudes Questionnaire): AUT (Attitudes to Unfamiliar Touch). ChT (Childhood Touch); variables are mean-centered

F(8.66)=0.77. p=.634

Table S5. Multiple regression analysis for overall intensity in the affective touch condition as the criterion

	R^2	B	SE	t	P	95% Confidence Interval for B LB	UB
	.09						
Intercept		45.98	3.45	13.34	<.001	39.09	52.86
Age		0.17	0.35	0.49	.627	-0.53	0.87
Sympathy		0.22	0.19	1.13	.262	-0.16	0.59
Condition order		8.09	4.87	1.66	.101	-1.63	17.81
TEAQ_AUT		2.65	2.97	0.89	.374	-3.27	8.58
TEAQ_ChT		1.66	2.83	0.59	.559	-3.99	7.31
BSI-18 anxiety		0.40	0.75	0.53	.596	-1.09	1.89
BSI-18 somatization		0.06	0.67	0.09	.928	-1.27	1.40
BSI-18 depression		0.05	0.58	0.08	.938	-1.12	1.21

Note. Intensity ratings averaged across six ratings; sympathy with toucher was measured during the affective touch condition; Condition order: if the nonaffective condition was completed first. this was coded with 0/ reversed order: 1; BSI (Brief Symptom Inventory); TEAQ (Touch Experiences and Attitudes Questionnaire): AUT (Attitudes to Unfamiliar Touch). ChT (Childhood Touch); variables are mean-centered

$F(8.66)=0.80$. $p=.604$

Table S6. Multiple regression analysis for overall intensity in the nonaffective touch condition as the criterion

	R^2	B	SE	t	P	95% Confidence Interval for B LB	UB
	.06						
Intercept		54.38	3.05	17.82	<.001	48.28	60.47
Age		0.05	0.30	0.15	.881	-0.56	0.65
**Sympathy**		0.01	0.18	0.07	.946	-0.34	0.37
**Condition order**		8.44	4.35	1.94	.057	-0.25	17.13
TEAQ_AUT		1.31	2.60	0.51	.615	-3.87	6.50
**TEAQ_ChT**		-0.65	2.48	-0.26	.793	-5.60	4.30
BSI-18 anxiety		-0.17	0.65	-0.26	.798	-1.46	1.13
**BSI-18 somatization**		0.13	0.58	0.23	.818	-1.03	1.30
**BSI-18 depression**		0.09	0.52	0.18	.860	-0.94	1.13

Note. Intensity ratings averaged across six ratings; sympathy with toucher was measured during the nonaffective touch condition; Condition order: if the nonaffective condition was completed first. this was coded with 0/ reversed order: 1; BSI (Brief Symptom Inventory); TEAQ (Touch Experiences and Attitudes Questionnaire): AUT (Attitudes to Unfamiliar Touch). ChT (Childhood Touch); variables are mean-centered

$F(8.66)=0.54$. $p=.826$

Table S7. Multiple regression analysis for the intensity change in the affective touch condition as the criterion

	R^2	B	SE	t	P	95% Confidence Interval for B LB	UB
	.03						
Intercept		-4.28	4.05	-1.06	.295	-12.38	3.81
Age		-0.30	0.41	-0.74	.464	-1.12	0.52
Sympathy		0.17	0.22	0.74	.460	-0.28	0.61
Condition order		0.13	5.72	0.02	.982	-11.29	11.56
TEAQ_AUT		-0.34	3.49	-0.10	.923	-7.31	6.63
TEAQ_ChT		-2.74	3.33	-0.82	.414	-9.39	3.91
BSI-18 anxiety		0.49	0.88	0.56	.578	-1.26	2.25
BSI-18 somatization		-0.77	0.79	-0.97	.334	-2.34	0.80
BSI-18 depression		0.06	0.68	0.09	.927	-1.30	1.43

Note. the criterion is calculated by subtracting the reported intensity after 100 seconds from the reported intensity after 600 seconds; sympathy with toucher was measured during the affective touch condition; Condition order: if the nonaffective condition was completed first. this was coded with 0/ reversed order: 1; BSI (Brief Symptom Inventory); TEAQ (Touch Experiences and Attitudes Questionnaire): AUT (Attitudes to Unfamiliar Touch). ChT (Childhood Touch); variables are mean-centered.

F(8.66)=0.24. p=.982

Table S8. Multiple regression analysis for the intensity change in the nonaffective touch condition as the criterion

	R^2	B	SE	t	P	95% Confidence Interval for B LB	UB
	.09						
Intercept		-10.75	4.44	-2.42	.018	-19.62	-1.88
Age		-0.16	0.44	-0.36	.721	-1.04	0.73
Sympathy		0.07	0.26	0.28	.781	-0.44	0.59
Condition order		2.46	6.34	0.39	.699	-10.19	15.12
TEAQ_AUT		-1.71	3.78	-0.45	.652	-9.27	5.84
TEAQ_ChT		-4.08	3.61	-1.13	.262	-11.29	3.12
BSI-18 anxiety		1.44	0.95	1.52	.133	-0.45	3.33
BSI-18 somatization		-0.12	0.85	-0.14	.892	-1.81	1.58
BSI-18 depression		-1.70	0.75	-2.25	.028	-3.20	-0.19

Note. the criterion is calculated by subtracting the reported intensity after 100 seconds from the reported intensity after 600 seconds; sympathy with toucher was measured during the nonaffective touch condition; Condition order: if the nonaffective condition was completed first. this was coded with 0/ reversed order: 1; BSI (Brief Symptom Inventory); TEAQ (Touch Experiences and Attitudes Questionnaire): AUT (Attitudes to Unfamiliar Touch). ChT (Childhood Touch); variables are mean-centered.

F(8.66)=0.81. p=.599

Table S9. Multiple regression analysis for the variance of pleasantness in the affective touch condition as the criterion

	R^2	B	SE	t	P	95% Confidence Interval for B LB	UB
	.14						
Intercept		75.44	24.33	3.10	.003	26.86	124.02
Age		1.45	2.46	0.59	.557	-3.46	6.36
Sympathy		-0.25	1.34	-0.18	.854	-2.92	2.43
Order		99.23	34.35	2.89	.005	30.65	167.81
TEAQ_AUT		10.36	20.95	0.49	.622	-31.46	52.19
TEAQ_ChT		29.37	19.99	1.47	.146	-10.54	69.27
BSI-18 anxiety		-1.19	5.28	-0.23	.822	-11.73	9.34
BSI-18 somatization		2.62	4.72	0.56	.580	-6.80	12.04
BSI-18 depression		2.84	4.10	0.69	.492	-5.36	11.03

Note. The criterion is the sum of the squared changes from one measurement time to the next divided by the number of changes; sympathy with toucher was measured during the affective touch condition; Condition order: if the nonaffective condition was completed first. this was coded with 0/ reversed order: 1; BSI (Brief Symptom Inventory); TEAQ (Touch Experiences and Attitudes Questionnaire): AUT (Attitudes to Unfamiliar Touch). ChT (Childhood Touch); variables are mean-centered.

$F(8.66)=1.33$. $p=.243$
